# Work–family conflict, financial issues and their association with self-reported health complaints among ready-made garment workers in Bangladesh: a cross-sectional study

**DOI:** 10.1007/s00420-022-01942-9

**Published:** 2022-12-08

**Authors:** Annegret Dreher, Rita Yusuf, Hasan Ashraf, Syed A K Shifat Ahmed, Christian Strümpell, Adrian Loerbroks

**Affiliations:** 1 Institute of Occupational, Social, and Environmental Medicine, Centre for Health and Society, Faculty of Medicine, University of Düsseldorf, Düsseldorf, Germany; 2 International Center for Biotechnology and Health (ICBH), Center for Health Population and Development (CHPD), Independent University, Dhaka, Bangladesh; 3 Department of Anthropology, Jahangirnagar University, Dhaka, Bangladesh; 4 Institute of Social and Cultural Anthropology, University of Hamburg, Hamburg, Germany

**Keywords:** Bangladesh, Ready-made garments, Work–family conflict, Financial strain, Poisson regression, Self-reported health

## Abstract

**Objectives:**

This study aims to quantify the degree of work–family conflict (WFC) and financial issues among ready-made garment (RMG) workers in Bangladesh and to investigate their potential associations with self-reported health outcomes.

**Methods:**

We conducted a cross-sectional survey among 1118 RMG workers in labor colonies in Dhaka, Bangladesh, in February and March 2021. Descriptive analyses were performed to characterize WFC (i.e., family life disturbing the job or facing problems in family due to the job) and financial issues (i.e., savings, debt, financial obligations, financial support). We ran multivariable Poisson regression models to examine possible associations between WFC and financial issues and workers’ health (self-reported general health and 10 specific health complaints).

**Results:**

We found low levels of WFC, low levels of savings, moderate levels of debt, and high levels of financial obligations: virtually all workers agreed they had to keep their job to financially support their spouse, children or other relatives. Only about a third of workers expected they would be able to receive financial support in case of a job loss. Work–family conflict was positively associated with poor health but not consistently with specific symptoms. Financial support was negatively associated, whereas being indebted was weakly positively associated with poor health.

**Conclusions:**

Our findings suggest low levels of WFC among RMG workers but high levels of financial obligations. Work–family conflict was positively associated with poor health, but not consistently with specific symptoms. Being indebted was weakly positively associated with poor health. Future prospective studies are needed to confirm these findings.

**Supplementary Information:**

The online version contains supplementary material available at 10.1007/s00420-022-01942-9.

## Background

The ready-made garment (RMG) industry in Bangladesh is among the world’s largest, with an estimated export value of 35.8 billion US$ in 2021, and employs over 4 million workers (Haque and Bari 2021; Bangladesh Garment Manufacturers and Exporters Association 2022). The industry produces almost exclusively for European and North American markets (Bangladesh Garment Manufacturers and Exporters Association 2022). Working conditions of RMG workers in Bangladesh are typically characterized by long working hours (Rahman and Nasrin 2016), poor salaries (Butler 2019; Ashraf 2017b), unfavorable working postures (Akhter et al. 2019; Ashraf 2017b), noise exposure (Akhter et al. 2019), emotional and sexual abuse (Naved et al. 2018), and workplace violence (Gibbs et al. 2019). In addition to these stressors, RMG workers are at high risk of workplace accidents. Among the most severe events in this respect, were the Tazreen Fashion factory fire in 2012 and the Rana Plaza collapse in 2013. More than 1250 workers were killed in these incidents, and survivors had to deal with severe injuries, unemployment and subsequent poverty, social stigma, and severe mental health issues (Kabir et al. 2021; Shovon 2020).

Several studies report high work stress levels among garment workers in Bangladesh (Steinisch et al. 2013; Parvin et al. 2018) and among RMG workers in other countries (Wang et al. 2010; Nagaraj et al. 2019). However, ethnographic and qualitative research from Bangladesh has also highlighted key stressors among RMG workers that exist beyond the context of their immediate workplace (Naved et al. 2018; Akhter et al. 2017a; Paul-Majumder 1996). These stressors include factors at the interface between work life and family life or stressors that are consequences of poor working conditions that also affect workers’ private life. Examples of such stressors are work–family conflicts (WFC) and financial issues (including, e.g., financial obligations for family members or debt) (Akhter et al. 2017a; Naved et al. 2018).

Work–family conflict describes the situation in which an individual experiences competing role expectations with respect to work and family (Schonfeld 2017). More specifically, three types of conflict have been described in literature (Schonfeld 2017): first, a time-based conflict arises when an individual’s time spent in one role interferes with fulfilling the other role. Second, a strain-based conflict arises when strain experienced in one role interferes with fulfilling the other role (e.g., being more irritable at home after a long day of work). Lastly, a behavior-based conflict arises when two roles require different behaviors (e.g., directive behaviors from work are not appropriate at home). All of these conflicts can manifest in two directions, with expectations and demands from work being incompatible with family expectations and demands or vice versa.

Past research has not only demonstrated a link between WFC and work outcomes (e.g., reduced work performance, poorer job satisfaction, and turnover intention (Amstad et al. 2011)), but also family outcomes (e.g., less marital satisfaction, poorer satisfaction with family life (Amstad et al. 2011)) and health outcomes (e.g., psychological distress (Nohe et al. 2015), increased risk of depressive and anxiety disorders (Grzywacz and Bass 2003), and increased blood pressure (Shockley and Allen 2013)).

Regarding WFC among RMG workers, a qualitative study by (Akhter et al. 2017a) described how female RMG workers from Bangladesh suffered from being separated from their children and how this caused stress and anxiety. Another qualitative study from Bangladesh highlighted that female workers felt tired and exhausted after working days and lacked energy for household tasks their husbands expected them to complete (e.g., childcare, food preparation, washing, and cleaning) (Akhter et al. 2019) (also see Karim 2022). One ethnographic study conducted among garment workers in India explained how strict shift schedules and movement restrictions at work interfered with workers’ private obligations (e.g., collecting water from municipal taps that were open only at unpredictable times) (De Neve 2014). Finally, sexual harassment at work may cause tensions between female RMG workers and their husbands. Female RMG workers are frequently accused of being sexually promiscuous and of having secret relationships with male co-workers (Naved et al. 2018), which may prevent them from disclosing harassment incidents toward their husbands.

To date, however, only one study has attempted to statistically quantify the burden of WFC among RMG workers in Bangladesh. In that study over 90% of workers stated that their job and family interfered with one another. Yet, the study also suggests that occupational obligations are more likely to interfere with family obligations than vice versa (Chowdhury et al. 2015). These findings suggest that prevention approaches and interventions may need to address the work context first rather than the family context. Nevertheless, evidence on WFC among RMG workers remains scarce and, to date, no study has investigated possible associations between WFC and health outcomes among RMG workers.

Further stressors for RMG workers concern financial matters. Such stressors are, for example, financial obligations for family members or being in debt. Financial strain has been linked to health outcomes, such as poor self-reported health (Artazcoz et al. 2021; Prentice et al. 2017), poor mental health (Steptoe et al. 2020), and poor sleep (Hall et al. 2009; Steptoe et al. 2020) across international studies. With respect to RMG workers in Bangladesh, qualitative research suggests that workers worry about not earning enough money to financially support their families (Akhter et al. 2017b). Workers in Bangladesh were also found to be burdened by the necessity of keeping their job to pay for family members or to pay off debts (Naved et al. 2018). A recent study by Yuan et al. (2022) was the first to quantify workers’ monthly income in Bangladesh, however, only providing categorized and no absolute data. The authors report nearly all RMG workers to be stressed due to their salary (Yuan et al. 2022). Beside this study, however, to the best of our knowledge, no studies have more broadly quantified financial issues (e.g., savings, debt) among RMG workers, nor have they linked these issues with workers’ health.

In summary, ethnographic and qualitative research suggests various stressors beyond the immediate workplace context among RMG workers. Stressors at the interface of work and private life have received only little attention in quantitative research to date. The first aim of this study was, consequently, to complement these findings by quantifying potential WFC and financial issues among garment workers in Bangladesh. Previous research has, furthermore, highlighted associations between occupational stressors of RMG workers and poor health (Steinisch et al. 2013; Shazzad et al. 2018; Hossain et al. 2018). Yet, no research has shed light on possible associations of WFC or financial issues with garment workers’ health. The second aim of this study, therefore, was to address this knowledge gap.

## Methods

### Study sample and procedure

We conducted a cross-sectional study in labor colonies situated in the Mirpur area of urban Dhaka, Bangladesh. This area is characterized by various adjacent garment-producing facilities, and we, therefore, assumed to find many garment workers living there. Fully structured face-to-face interviews were conducted by seven female interviewers of a professional survey company with participants who labeled themselves as garment workers. Interviews took place between February 20th and March 18th, 2021, in evening hours after garment workers’ working days or on days off. As this study was conducted during the COVID-19 pandemic, all interviewers were equipped with disinfectants and personal protective equipment.

Participants were selected at random according to the following procedure: together with local informants the survey team estimated the total number of households per colony and divided this number by the estimated number of garment workers per colony. The obtained number served as interval in which households were approached. Interviewers approached a randomly selected first household at the border of a colony and then moved on to the next household following the obtained interval number and so on. In cases where more than one self-labeled garment worker was met, one worker was selected at random for participation. In case this person declined, a second person from this household was approached and so on. Similar approaches have been applied in studies conducted in e.g., slum settings (Renzaho et al. 2017; Sharma and Panigrahi 2021).

All participants provided written consent. There was no financial compensation for participation. Self-labeled garment workers who were at least 18 years old were eligible to participate, regardless of their exact type of employment within a factory.

### Ethical approval

The Bangladesh Medical Research Council (BMRC) approved the study (Registration number 290 16 03 2020, Reference BMRC/NREC/2019–2022/705) as well as the ethics committee of the Medical Faculty of the University of Duesseldorf, Germany (study number 5427).

### Questionnaire

We used a self-developed questionnaire that measured not only sociodemographic characteristics of garment workers (e.g., age, sex, education, marital status, smoking status) but also information on WFC, financial matters, and self-reported health outcomes.

### Work–family conflict

*WFC* was assessed using the two items ‘Your family life has disturbed you in doing your job as good as you could do.’ (yes/no) and ‘Often you face problems in your family due to your job’ (yes/no). Item wordings were taken from (Chowdhury et al. 2015). However, the original 5-point Likert scale was reduced to a binary answer scale in this study based on experiences during a pilot study (see below).

### Financial situation

We inquired in detail about the financial situation of the workers. First, *financial obligations* were measured by two items ‘You must keep your current job, because you need to support your spouse or children financially.’ (yes/no) and ‘You must keep your job, because you need to financially support other relatives to a significant extent.’ (yes/no). Second, to describe the general financial situation of RMG workers we inquired about the *amount of savings*, *debt* and the *amount handed over to family members* (in Bangladeshi taka) as well as the *number of people depending on that wage*. Finally, *financial support* was assessed as follows: ‘Could you call on anyone to support you financially if you lost your job?’ (yes/no). Again, the scope of items was informed by prior ethnographic research (Ashraf 2017a; Chand 2006). As no established questionnaire items were available, item wordings were self-developed and subsequently piloted (see below).

### Health outcomes

We measured *self-reported general health* using the single item ‘In general, how would you rate your health?’ rated on a 5-point Likert Scale ‘Very good, good, moderate, bad, very bad’. This item has been found to correlate with physiological health markers (Warnoff et al. 2016; Gallagher et al. 2016) and to predict health status (Tseng et al. 2021) and mortality (Benyamini and Idler 1999; Inkrot et al. 2016).

We, furthermore, inquired about ten specific health complaints that had been examined in a previous study of our group among RMG workers (Steinisch et al. 2013) and have been informed by ethnographic research (Strümpell and Ashraf 2012; Ashraf 2017a). The occurrence of these health complaints (i.e., *back pain*, *sleeplessness*, *headache*, *breathing problems*, *cold*, *tuberculosis*, *jaundice*, *stomach problems*, *muscle cramps*, *eye problems*) within the last 12 months was measured binarily (yes/no).

### Translation and piloting of the final instrument

The questionnaire was developed in close cooperation among all members of the study team, which comprises significant expertise in the fields of epidemiology and cross-cultural questionnaire design (AL, AD, RY, SA), ethnographic research (CS and HA) as well as epidemiological study conduction in Bangladesh (RY and SA). The multidisciplinary nature of the team likely increased the instruments’ face validity. Translation from English into Bangla was performed by experts of a professional survey company. A subsequent back translation was performed by a bilingual assistant professor of public health. We discussed inconsistencies between translations until we reached a consensus on how to resolve them and how to refine the Bangla-language questionnaire.

The questionnaire was then piloted in a sample of 56 garment workers working at two garment factories in Uttara, Dhaka, (*n* = 50) and the adjacent district Mirpur (*n* = 6). Contact details of the pilot study participants were not recorded, thus it cannot be excluded that they also participated in the main study (solely conducted in Mirpur). After piloting, the instrument was shortened from 111 to 77 items and the response scale of questions was partially simplified for comprehension reasons, leading to the binary response format mostly applied in this study. The original study protocol was changed after the pilot study due to the SARS-CoV-2 pandemic, which made data collection in factories (as originally planned) impossible. Changes were also made in terms of the reduction of the number of questionnaire items and the changed answer format.

### Statistical analysis

Only garment workers who stated to have mainly worked in the garment sector in the past three months were included in the analyses (*n* = 1118). This criterion was used to maximize the likelihood that participants had experienced a reasonable period of exposure to the working conditions in RMG factories. Descriptive analysis was performed to display sociodemographic characteristics and to characterize the burden of WFC, financial matters, and health complaints using absolute and relative frequencies or means and standard deviations.

To investigate possible associations of WFC and financial matters with self-reported health and health complaints, we ran robust Poisson regression models (Knol et al. 2012) using IBM SPSS 27, which estimate prevalence ratios (PRs) with 95% confidence intervals (CI). Independent variables for *WFC* were ‘family life has disturbed job’ (yes vs. no) and ‘facing problems in family due to job’ (yes vs. no).

With respect to financial issues, independent variables were ‘financially supporting children or spouse’ (yes vs. no), ‘financially supporting other relatives’ (yes vs. no), the number of persons depending on one’s wage (continuous numerical value), and ‘financial support available’ (yes vs. no). The inquired amounts of savings and debt were, first, dichotomized into variables ‘any savings’ (yes vs. no) and ‘any debt’ (yes vs. no). Second, the amounts of savings and debt were compared to participants’ individual income. The resulting variables ‘*amount of savings’* and ‘*amount of debt’* referred to the amount of savings/debt of ‘less than 1 monthly salary’, ‘1–2 monthly salaries’, or ‘3 or more monthly salaries’.

*Self-reported general health* (poor vs. good) and self-reported health complaints (yes vs. no) served as outcome variables. Self-reported health was dichotomized (‘very good’/‘good’ health vs. ‘moderate’/’bad’/’very bad’ health) in accordance with prior research from our group (Steinisch et al. 2013). As mentioned above, we measured a total of 10 health complaints; however, two of them with prevalences below 10% (i.e., tuberculosis and breathing problems) were excluded from the analyses due to possible instability of our statistical models and due to their assumingly lesser relevance to RMG workers.

All independent variables were analyzed in separate models adjusted for age, sex, marital status, education, and smoking. As previous research in the RMG sector has suggested that not only WFC but also financial obligations vary with the age and sex of workers (Chowdhury et al. 2015; Ashraf 2017a), we ran additional analyses stratified by sex and age (using the median split at 26 years as a cutoff). To test for the significance of sex and age differences, we included an additional interaction term in Poisson regression models (stressor*sex and stressor*age, respectively). The results of all additional analyses are presented as additional file (supplementary file 1).

## Results

Interviewers approached 4375 households, among these were 1827 households with garment workers (41.8%). Among these, 1264 workers participated in the survey of which 1118 reported to have mainly worked in the garment sector in the previous three months and were, therefore, included in the analyses. Participant characteristics can be found in Table 1. The majority of participants were female (71.3%), young (mean age 26.2 years, SD 7.1 years), married (66.0%), had children (59.5%), and had lower educational levels (64.1% education up to grade 5). Most participants rated their health as either very good or good (62.1%). The most prevalent self-reported health complaints were headache (68.3%), cold (55.3%), and back pain (50.7%).

**Table 1:** Sociodemographic characteristics of *n* = 1118 ready-made garment workers

Characteristics	Total *n* (%)	Women only *n* (%)	Men only *n* (%)	Younger group^a^ *n* (%)	Older group^a^ *n* (%)
Sex					
Female	797 (71.3)	797 (100)	–	433 (71.0)	364 (71.7)
Male	321 (28.7)	–	321 (100)	177 (29.0)	144 (28.3)
Age, mean (SD)	26.2 (7.2)	26.3 (7.4)	26.1 (6.9)	21.0 (2.6)	32.5 (6.0)
Highest level of education					
No formal education	190 (17.0)	155 (19.4)	35 (10.9)	58 (9.5)	132 (26.0)
Grade 1–5	527 (47.1)	384 (48.2)	143 (44.5)	292 (47.9)	235 (46.3)
Grade 6–10	330 (29.5)	219 (27.5)	111 (34.6)	213 (34.9)	117 (23.0)
Lower secondary exam (matric/SSC)	47 (4.2)	28 (3.5)	19 (5.9)	33 (5.4)	14 (2.8)
Higher secondary exam (Intermediate/HSC)	20 (1.8)	8 (1.0)	12 (3.7)	14 (2.3)	6 (1.2)
Bachelor’s degree	3 (0.3)	2 (0.3)	1 (0.3)	0 (0.0)	3 (0.6)
Postgraduate degree	1 (0.1)	1 (0.1)	0 (0.0)	0 (0.0)	1 (0.2)
Marital status					
Married	738 (66.0)	524 (65.7)	214 (66.7)	313 (51.3)	425 (83.7)
Separated or divorced	64 (5.7)	60 (7.5)	4 (1.2)	27 (4.4)	37 (7.3)
Never married	281 (25.1)	178 (22.3)	103 (32.1)	267 (43.8)	14 (2.8)
Husband/wife died	35 (3.1)	35 (4.4)	0 (0.0)	3 (0.5)	32 (6.3)
Has children					
Yes	665 (59.5)	510 (64.0)	155 (48.3)	208 (34.1)	457 (90.0)
No	453 (40.5)	287 (36.0)	166 (51.7)	402 (65.9)	51 (10.1)
Working hours per day in current job, mean (SD)	10.2 (1.6)	10.3 (1.6)	10.3 (1.6)	10.3 (1.6)	10.3 (1.6)
Currently smoking tobacco products					
Yes	279 (25.0)	146 (18.3)	133 (41.4)	94 (15.4)	185 (36.4)
No	839 (75.0)	651 (81.7)	188 (58.6)	516 (84.6)	323 (63.6)
Self-rated health					
Very good	198 (17.7)	130 (16.3)	68 (21.2)	125 (20.5)	73 (14.4)
Good	496 (44.4)	337 (42.3)	159 (49.5)	291 (47.7)	205 (40.4)
Moderate	331 (29.6)	251 (31.5)	80 (24.9)	156 (25.6)	175 (34.4)
Bad	72 (6.4)	61 (7.7)	11 (3.4)	31 (5.1)	41 (8.1)
Very bad	21 (1.9)	18 (2.3)	3 (0.9)	7 (1.1)	14 (2.8)
Prevalence of self-reported health complaints in the past 2 months					
Back pain	567 (50.7)	433 (54.3)	134 (41.7)	292 (47.9)	275 (54.1)
Sleeplessness	343 (30.7)	246 (30.9)	97 (30.2)	153 (25.1)	190 (37.4)
Headache	764 (68.3)	571 (71.6)	193 (60.1)	413 (67.7)	351 (69.1)
Breathing problems	74 (6.6)	65 (8.2)	9 (2.8)	32 (5.2)	42 (8.3)
Cold	618 (55.3)	457 (57.3)	161 (50.2)	324 (53.1)	294 (57.9)
Tuberculosis	9 (0.8)	7 (0.9)	2 (0.6)	3 (0.5)	6 (1.2)
Jaundice	497 (44.5)	363 (45.5)	134 (41.7)	259 (42.5)	238 (46.9)
Stomach problems	323 (28.9)	244 (30.6)	79 (24.6)	160 (26.2)	163 (32.1)
Muscle cramps	477 (42.7)	363 (45.5)	114 (35.5)	234 (38.4)	243 (47.8)
Eye problems	253 (22.6)	194 (24.3)	59 (18.4)	105 (17.2)	148 (29.1)

*SD* standard deviation

^a^According to median split at < 26/ ≥ 26 years

Prevalences of WFC and financial matters are presented in Table 2. We found fairly low levels of *WFC*, that is, 14.8% agreed that their family life had disturbed their job and 12.5% agreed that they faced problems in their family due to their job. As much as 23.7% of the RMG workers reported they had *any savings,* and mean saving were 8244 taka (roughly 94$ or 87€). When comparing workers’ savings to their individual monthly income, the majority of workers (81.4%) reported to have an *amount of savings* of less than one monthly salary. In total, 40.8% reported *any debt*, and mean debt amount was 16,641 taka (roughly 193$ or 175€). Again, for the majority of workers (69.2%) the *amount of debt* equaled less than one monthly salary.

**Table 2:** Prevalences of work–family conflict and financial matters among *n* = 1118 ready-made garment workers

Characteristics	Total *n* (%)	Women only *n* (%)	Men only *n* (%)	Younger group** *n* (%)	Older group** *n* (%)
Work–Family Conflict					
Family life has disturbed doing job	165 (14.8)	123 (15.4)	42 (13.1)	88 (14.4)	77 (15.2)
Facing problems in family due to job	140 (12.5)	108 (13.6)	32 (10.0)	74 (12.1)	66 (13.0)
Savings					
Has any savings	265 (23.7)	193 (24.2)	72 (22.4)	141 (23.1)	124 (24.4)
Amount of savings in taka, mean (SD)	8244 (20427)	8664 (20,966)	7200 (19,012)	7515 (19,134)	9118 (21,865)
Amount of savings related to individual income					
Less than 1 monthly salary	910 (81.4)	640 (80.9)	270 (84.1)	503 (82.7)	407 (80.8)
1–2 monthly salaries	90 (8.1)	62 (8.0)	27 (8.4)	45 (7.4)	45 (8.9)
3 or more monthly salaries	112 (10.1)	88 (11.1)	24 (7.5)	60 (9.8)	52 (10.2)
Debt					
Has any debt	456 (40.8)	318 (39.9)	138 (43.0)	204 (33.4)	252 (49.6)
Amount of debt in taka, mean (SD)	16,641 (34863)	16,664 (35093)	16,584 (34,340)	9520 (23,538)	25,192 (43,336)
Amount of debt related to individual income					
Less than 1 monthly salary	769 (69.2)	547 (69.2)	222 (69.2)	471 (77.5)	298 (59.1)
1–2 monthly salaries	137 (12.3)	88 (11.1)	49 (15.3)	68 (11.1)	69 (13.6)
3 or more monthly salaries	206 (18.4)	156 (19.7)	50 (15.6)	69 (11.3)	137 (27.0)
Wealth and income					
Monthly wage in taka, mean (SD)	10,240 (3347)	9459 (2910)	12,164 (3572)	9641 (3203)	10,963 (3376)
Savings minus debt in taka, mean (SD)	− 8398 (41,320)	− 8000 (41,975)	− 9384 (39,682)	− 2005 (31,021)	− 16,073 (49,973)
Financial obligations					
Must keep job for financial support of spouse/children	803 (95.3)^a^	587 (94.5)^a^	216 (97.3)^a^	327 (92.9)^a^	476 (96.9)^a^
Must keep job for financial support of other relatives to significant extent	696 (62.3)	449 (56.3)	247 (76.9)	440 (72.1)	256 (50.4)
Number of people depending on wage, mean (SD)	4.1 (1.5)	3.9 (1.4)	4.4 (1.5)	3.9 (1.5)	4.2 (1.4)
Amount of your wage handed over to family members in taka, mean (SD)	6308 (4409)	5800 (4143)	7499 (4779)	6388 (3908)	6179 (4936)
Financial support					
Could you call on anyone to support you financially if you lost your job?	393 (35.2)	282 (35.4)	111 (34.6)	280 (45.9)	113 (22.2)

*SD* standard deviation

^a^Only for RMG workers with spouse or children

^b^according to median split at 26 years

Subtracting debt from savings showed that RMG workers on average are in debt (mean wealth after subtracting debt from savings − 8398 taka, reflecting roughly 97$ or 91€). Virtually all RMG workers (95.3%) agreed they had to keep their job to support their spouse or children and almost two-thirds said they also had to keep their job to financially support other relatives. The mean number of people depending on a workers’ wage was reported to be 4.1 (SD 1.5). The mean amount of wage handed over to family members per month was 6,308 taka (roughly 71$ or 67€). Only about one-third of workers (35.2%) could call on anyone to financially support them if they lost their job.

Stratified analyses according to sex and age revealed similar values of WFC across sex and age groups. The monthly mean wage of male workers was about 30% higher than the mean wage of female workers (12,164 taka (about 130$ or 124€) vs. 9459 taka (about 102$ or 96€)). Women had slightly more savings than men. The amount of debt was similar between men and women. Notably, older workers reported to have much more debt than younger workers (mean debt 25,192 taka (about 270$ or 257€) vs. 9520 taka (about 102€ or 97€).

Men were more likely than women to report to support other relatives than their spouse or children to a significant extent. In accordance with this, men handed over greater amounts of money to family members than women, and a larger number of people depended on their wage. In terms of share of income, however, men and women reported to hand over the same percentage of their wage, that is about 61%, to their families. When divided by median age, younger RMG workers more frequently reported to have to financially support other relatives than spouse/children. Lastly, financial support also largely differed between age groups to the extent that younger workers were more often able to call on anyone.

Regarding possible associations between WFC and financial matters with self-reported health outcomes, we generally found weak and only partly significant associations (see Table 3). The only meaningful associations with poor self-reported health (SRH) were observed for family life having disturbed workers’ job (PR 1.36 [95%CI 1.14–1.62]) and the availability of financial support (PR 0.76 [95%CI 0.63–0.90]). Most other investigated health outcomes were also positively associated with family life having disturbed workers’ job and negatively associated with the availability of financial support. Being indebted also showed a constant positive association with all health outcomes, albeit not constantly significant.

**Table 3:** Association of work–family conflict and financial matters with self-reported health complaints among *n *= 1118 ready-made garment workers

	Poor SRH		Back pain		Sleeplessness		Headache		Cold	
	PR	95% CI	PR	95% CI	PR	95% CI	PR	95% CI	PR	95% CI
Family life has disturbed job (yes vs. no)	**1.36**	**1.14–1.62**	1.14	0.99–1.32	***1.29***	**1.04–1.61**	1.03	0.92–1.15	1.13	0.99–1.30
Problems in family due to job (yes vs. no)	1.10	0.89–1.35	1.16	0.99–1.35	1.16	0.91–1.48	1.04	0.93–1.16	1.11	0.96–1.29
Financial support available (yes vs. no)	**0.76**	**0.63–0.90**	**0.86**	**0.76–0.98**	**0.73**	**0.60–0.90**	**0.89**	**0.81–0.97**	**0.88**	**0.78–0.99**
Any savings (yes vs. no)	0.93	0.78–1.11	1.10	0.97–1.25	0.97	0.79–1.20	**1.09**	**1.00–1.19**	1.01	0.90–1.14
Any debt (yes vs. no)	1.13	0.97–1.31	**1.17**	**1.04–1.31**	**1.21**	**1.01–1.45**	1.09	1.01–1.18	**1.15**	**1.03–1.28**
Amount of savings										
Less than 1 monthly salary	Ref.	Ref.	Ref.	Ref.	Ref.	Ref.	Ref.	Ref.	Ref.	Ref.
1–2 monthly salaries	1.02	0.78–1.34	1.02	0.83–1.24	1.19	0.90–1.58	1.00	0.87–1.16	1.06	0.89–1.27
3 or more monthly salaries	0.84	0.65–1.09	1.13	0.95–1.34	0.88	0.64–1.20	1.08	0.96–1.22	0.94	0.78–1.13
Amount of debt										
Less than 1 monthly salary	Ref.	Ref.	Ref.	Ref.	Ref.	Ref	Ref.	Ref	Ref.	Ref.
1–2 monthly salaries	0.99	0.78–1.25	1.10	0.93–1.31	1.09	0.84–1.43	1.07	0.96–1.20	1.01	0.85–1.19
3 or more monthly salaries	1.07	0.89–1.29	**1.16**	**1.01–1.34**	1.20	0.97–1.50	**1.11**	**1.00–1.22**	**1.22**	**1.07–1.38**
Number of persons depending on wage	0.99	0.94–1.05	1.01	0.96–1.05	1.01	0.95–1.08	1.00	0.97–1.03	0.99	0.95–1.03
Having to financially support children or spouse (yes vs. no)	1.25	0.80–1.97	1.11	0.79–1.56	1.14	0.68–1.89	0.96	0.78–1.19	0.91	0.70–1.19
Having to financially support other relatives (yes vs. no)	0.88	0.75–1.03	1.02	0.90–1.16	1.00	0.82–1.21	0.92	0.84–1.01	0.92	0.82–1.04

	Jaundice		Stomach problems		Muscle cramp		Eye problems	
	PR	95% CI	PR	95% CI	PR	95% CI	PR	95% CI
Family life has disturbed job (yes vs. no)	1.08	0.90–1.29	1.02	0.79–1.32	**1.43**	**1.23–1.66**	1.16	0.87–1.55
Problems in family due to job (yes vs. no)	1.06	0.87–1.28	1.15	0.88–1.49	**1.41**	**1.21–1.66**	1.18	0.87–1.60
Financial support available (yes vs. no)	0.89	0.77–1.03	**0.80**	**0.65–0.98**	0.86	0.74–1.01	**0.71**	**0.55–0.92**
Any savings (yes vs. no)	**0.83**	**0.71–0.98**	0.96	0.77–1.20	1.09	0.93–1.26	0.85	0.66–1.11
Any debt (yes vs. no)	**1.14**	**1.00–1.31**	**1.37**	**1.14–1.65**	**1.20**	**1.05–1.38**	1.28	1.03–1.59
Amount of savings								
Less than 1 monthly salary	Ref	Ref	Ref	Ref	Ref	Ref	Ref	Ref
1–2 monthly salaries	0.82	0.63–1.09	0.85	0.58–1.24	1.20	0.98–1.48	0.71	0.46–1.11
3 or more monthly salaries	0.90	0.72–1.14	0.92	0.67–1.26	0.95	0.75–1.20	1.00	0.71–1.41
Amount of debt								
Less than 1 monthly salary	Ref	Ref	Ref	Ref	Ref	Ref	Ref	Ref
1–2 monthly salaries	1.19	0.98–1.44	1.28	0.98–1.67	1.06	0.85–1.31	1.20 **1.4**	0.86–1.67
3 or more monthly salaries	**1.19**	**1.01–1.41**	**1.41**	**1.13–1.77**	**1.2**	**1.09–1.50**	**3**	**1.11–1.83**
Number of persons depending on wage	0.98	0.94–1.03	1.03	0.97–1.10	0.97	0.92–1.02	**1.12**	**1.04–1.21**
Having to financially support children or spouse (yes vs. no)	1.10	0.75–1.61	0.89	0.55–1.42	1.29	0.83–2.00	1.70	0.88–3.26
Having to financially support other relatives (yes vs. no)	1.02	0.88–1.18	0.87	0.71–1.07	1.09	0.93–1.27	1.16	0.91–1.47

Poisson regression results in form of prevalence ratios (PR) with respective 95% confidence intervals (CI). Adjusted for age, sex, marital status, education and tobacco use. Significant findings highlighted in bold. *SRH* self-reported health

Results of stratified association analysis according to sex revealed several differences between men and women (see supplementary file 1). However, only few of these were statistically significant and no clear pattern emerged. After stratifying association analyses according to age (at the median split), several significant differences between the older and younger group of workers emerged. These suggested stronger associations between WFC and financial matters with health outcomes for the older age group (see supplementary file 1).

## Discussion

Our study is the first to provide insights into RMG workers’ financial situation. Whereas previous studies have highlighted a burden of financial constraints and obligations, no studies have provided specific numbers on, e.g., savings, debt, and the amount of money handed over to family members so far. Our study, furthermore, adds to the scare research on WFC among RMG workers by providing data collected during the COVID-19 pandemic for the first time. Lastly, this is also the first study to analyze possible associations of both concepts, WFC and financial issues, with health outcomes. We found rather low levels of WFC, high levels of financial obligations toward either one’s spouse and children or other relatives and moderate levels of financial support when needed. While only slightly more than 20% of workers had savings, over 40% reported to be indebted. Work–family conflict was positively associated with poor health, but there was no consistent relationship with specific symptoms. Financial support was negatively and being indebted was weakly positively associated with poor health.

### Results from descriptive analyses

Our results suggest relatively low levels of WFC among RMG workers (i.e., 14.8% agreed that their family life had disturbed their job, 12.5% agreed that they faced problems in their family due to their job). This is in contrast with findings by Chowdhury et al. (2015), who found not only much higher WFC levels among RMG workers in Bangladesh but also a different direction of conflict: they found that workers were more likely to face problems in their family due to their job (71.9%) than vice versa (26.5%). Both our study and the study by Chowdhury et al. (2015) were conducted among garment workers in Bangladesh using the same questionnaire items, albeit with a simplified answer scale in our study. We surveyed a random sample of garment workers in labor colonies, whereas Chowdhury et al. (2015) surveyed a convenience sample of workers in factories. Nevertheless, it is unlikely that these differences in methodology fully explain the large differences in WFC observed.

A possible explanation for the low degree of WFC in this study may be that 40% of the participants did not have children and, therefore, had no childcare obligations. Likewise, 34% of the participants in our sample were not married and, therefore, likely did not experience additional demands from their spouse and in-laws. The information on marital status and on whether workers had children was not provided in the study by Chowdhury et al. (2015). A second potential explanation for the differing findings is that our study was conducted more recently than the study by Chowdhury et al. (2015), that is, during the COVID-19 pandemic at a time after garment factories had reopened after large lockdown-related shutdowns. This may have altered workers’ attitudes toward their jobs to that effect that they might have seen work more positively out of relief of just being employed again. However, a study by Islam et al. (2022) reported increased financial pressure on female RMG workers from their husbands who lost their jobs during the COVID-19 pandemic (Islam et al. 2022). This in turn could have increased WFC levels.

Only about 23% of workers reported to have savings, leaving 77% of workers without any savings. This number is almost twice as high as previously found in a study conducted by (Naved et al. 2018), who only found that 40% of RMG workers did not have any savings. The mean saving amount in our study was 8244 taka (roughly 96$ or 91€), which corresponds to about one month of minimum income (the legal minimum wage of garment workers in Bangladesh was set to 8000 taka per month in 2018 (United News of Bangladesh 2018)). In their study, Naved et al. (2018) found that over 38% of RMG workers reported savings of 20,000 taka or more. In our study, however, only half as many workers (15.1%) had savings of 20,000 or above. Our study was conducted during the COVID-19 pandemic, which may have greatly contributed to the lower amounts of savings observed.

Literature describes, for example, severe pay cuts for Bangladeshi workers in general (HRB and Chowdhury Center for Bangladesh studies at UC Berkeley 2021) or complete loss of income for RMG workers (HRB and Chowdhury Center for Bangladesh studies at UC Berkeley 2021; LeBaron et al. 2021; Glover 2020; The Daily Star 2020) due to the pandemic. These emanated from the global supply chain as international retailers broke binding contracts with garment factories and refused to pay for produced garments laying in ports or stores (Siddiqi 2022, 2020). Garment factories, in turn, had to pay for all arising expenses until shipment of garments (including workers’ salaries) and suddenly could no longer pay salaries, which led to immediate dismissals and labor law violations (Siddiqi 2020).

In line with this, Munim et al. (2022) report that maintaining employees’ salaries as before COVID-19 was the least frequently implemented measure among all measures implemented by RMG industry experts in Bangladesh during the COVID-19 pandemic (Munim et al. 2022). The abovementioned pay cuts may have led to fewer possibilities of saving money and were paralleled by a sudden inflation of food and transportation costs during the pandemic (Rahman and Matin 2022, 2021) and the consequent need to spend savings to cover living costs. Likewise, the number of RMG workers without any savings rose by 25% in the course of the pandemic (LeBaron et al. 2021).

Our results, furthermore, suggest that over 40% of workers are indebted. The mean debt amount was 16,641 taka (roughly 193$ or 184€), which is equivalent to about two months of minimum income. Incurring debts was a prominent coping strategy for Bangladesh’s general population during the COVID-19 crisis as cost for necessities rose (Rahman et al. 2020), especially for female garment workers whose husbands lost their jobs in the informal sector during the crisis (Islam et al. 2022). A report among garment workers in Ethiopia, Myanmar, India, and Honduras suggests that during the COVID-19 pandemic debt levels increased by an average of 16% and workers were having difficulties paying back their loans (LeBaron et al. 2021) (also see Hewamanne and Yadav 2022). Unfortunately, published numbers on garment workers’ household debt before the pandemic were not available for comparison.

The majority of workers agreed they had to keep their job to financially support their spouse, children, or other relatives. This is in accordance with reports from ethnographic and qualitative studies among RMG workers (Akhter et al. 2017b; Chand 2006; Naved et al. 2018). Roughly, one-third of RMG workers could call on someone to support them if they lost their job. This degree of financial support is much lower than compared to studies conducted in the USA (Fingerman et al. 2009) or Sweden (Fritzell and Lennartsson 2005). Possibly, relatives and friends of RMG workers suffered from financial strains themselves and, therefore, may not have been able to lend money. Financial support is, however, of special interest considering many garment workers have lost their jobs during the COVID-19 pandemic (LeBaron et al. 2021).

With respect to stratified analyses according to sex, men more frequently had to financially support relatives other than their children or spouse, gave a larger absolute share of their money to their families and more people depended on their wages. However, with respect to the overall salary, men and women handed out similar percentages of their earnings to family members (about 61%). Male workers’ monthly salary was about 30% higher than female workers’ mean salary. This discrepancy is likely partially explained by the different types of jobs performed by male and female workers within a factory. While female workers typically do manual labor (e.g., sewing machine operators), male workers more frequently work in management or supervisor positions.

With respect to stratification according to age, younger workers more frequently reported being able to call on someone in case financial support is needed than older workers. Accordingly, older workers reported much more debt than younger workers did. Possibly, this may be explained by the fact that older workers were more likely to have children compared to younger workers (90% vs. 34%), resulting in increased financial obligations.

### Results from association analyses

The results from our association analyses suggest WFC of RMG workers to be associated with poor health. Here, family-to-work conflict was more strongly and significantly associated with poor health than work-to-family conflict. It is conceivable that the double burden of family and work obligations leads to physical exhaustion and mental stress among garment workers, which both in turn may have a negative effect on health. Several international studies from, e.g., the US.A, Taiwan, Switzerland, and Norway have shed light on the association between WFC and poor overall employee health (Tsukerman et al. 2020; Pien et al. 2020; Hämmig and Bauer 2014), burnout (Pien et al. 2020), shorter sleep duration (Berkman et al. 2015), insomnia (Lee et al. 2019; Vedaa et al. 2016), musculoskeletal disorders (Hämmig and Bauer 2014), and metabolic syndrome indicators (Versey and Tan 2020). In their study among employees in Switzerland Hämmig and Bauer (2014) report that work–life conflict was the psychosocial working condition that was strongest linked to adverse health (compared to, e.g., low social support, low job autonomy).

We found that having savings was associated with better health, albeit not significantly. As discussed earlier, mean savings in our study sample were as much as a month’s salary. This amount may be quickly used up, e.g., in case of dismissal and might, therefore, not be a significant relief for workers. During the COVID-19 pandemic, research, furthermore, suggested many Bangladeshis to have relied on their savings to compensate rising food costs (Rahman et al. 2020). However, savings may not only be viewed as a relief but also as a burden for garment workers. Research among female RMG workers in Bangladesh suggested that having savings beyond a certain threshold increased the likelihood of workers to experience violence at home (Naved et al. 2018). The authors of the study explained this by a likely increase in arguments over access to the saved money and over how to spend it.

Debt in our study was, in contrast, significantly associated with poor health. The link between financial strain and poor health is well-established in the international literature (Artazcoz et al. 2021; Prentice et al. 2017; Steptoe et al. 2020). Several hypotheses have been put forward to explain this link: lower financial resources may decrease access to healthcare, utilization of healthcare services and treatment adherence (Grafova 2018b). Financial strain may also result in a feeling of uncertainty about financial resources leading to elevated stress levels, which then result in poorer health (Grafova 2018a). This is in line with a longitudinal study that suggests only a small contribution of health-related behaviors to the association between financial strain and health. Instead, it suggests biological pathways to explain the association (Prentice et al. 2017).

Being indebted was weakly positively associated with poor health. Incurring debt was a prominent coping strategy for Bangladeshis during the COVID-19 pandemic (Rahman et al. 2020). Considering RMG workers’ low salaries, however, it may be impossible for workers to pay back their debt (Islam et al. 2022), which may result in a high stress level and possibly poor health. International research across different populations also suggests that being indebted is associated with several health complaints, such as sleep problems (Hall et al. 2008; Warth et al. 2019), back pain (Ochsmann et al. 2009), and chronic disease (Blomgren et al. 2016).

No other meaningful associations between financial matters and the health of RMG workers were observed in our data. Given the high levels of agreement with financial obligations, however, it is conceivable that the number of individuals who did not have financial obligations was too small to identify significant associations.

### Strengths and limitations

Our study has several strengths. First, the questionnaire was developed by an interdisciplinary research group and was translated, back-translated, and profoundly piloted, which greatly enhanced face validity and likely comprehensibility of the instrument. Second, we performed random sampling of garment workers, which reduced the likelihood of selection bias and increased the representativeness of the study population compared to the total population of RMG workers. Selection bias was additionally reduced by the fact that interviews were conducted in the evenings and on weekends outside working hours. We were able to recruit a large number of participants who most likely came from various factories and, therefore, had a great variety of working conditions and likely varying degrees of WFC and financial matters.

Nevertheless, some limitations need mentioning. We inquired about psychosocial working conditions and found weak to moderate associations with health outcomes. It is possible that physical working conditions play a greater role for workers’ health in the RMG setting. Due to the cross-sectional design of the study, the detected associations cannot necessarily be interpreted as causal. The exact response rate on the individual level cannot be determined and may be lower than the response rate on the household level (69.1%). Selection bias cannot be ruled out, as it is possible that workers with better working conditions were more likely to participate in the study. Furthermore, WFC and financial matters may have changed during the COVID-19 pandemic and may have not been representative compared to pre-pandemic times (i.e., we might have found higher values for, e.g., increase of financial demands or increased WFC (Islam et al. 2022)).

Our study is specific to Dhaka urban settings in one of the oldest export garment factory hubs (i.e., Mirpur). However, our data may not be representative of the entire RMG workforce in Bangladesh, including other large garment factory hubs, such as in Chittagong. Future research would be useful to understand how workers’ lives and health outcomes are unfolding in the newly developing export garment factory hubs outside Dhaka and in the export processing zones.

### Implications

Psychosocial working conditions have largely been absent in public and policy discourses in Bangladesh. The long and allegedly inherent persistence of adverse psychosocial working conditions may have brought about that they are perceived as an ‘assimilated phenomenon’ and thus attract less attention. Our study is an attempt to address this gap. The results of this study offer no immediate benefit to the participants. However, if confirmed by prospective studies, they may indirectly contribute to better working conditions in the long term. The insights into workers’ health help identify target areas for specific health-related interventions (e.g., against headache or back pain).

Our results suggest increasing workers’ salaries as a measure to potentially improve workers’ health. However, solely increasing salaries has been demonstrated counterproductive for workers’ health and consequently a profound change in working conditions is needed so that factory owners do not offset the increase in workers’ salaries by, e.g., shortening their breaks or increasing production targets (Kabir et al. 2022). Global labor platforms and national EU and North-American governments who are in bi-lateral trade and policy relations with Bangladesh can benefit from the findings of this work as industry stakeholders. The results of the present study are also interesting for factory owners, who should also have an interest in a healthy workforce, because healthy employees are generally more productive. Healthy employees may also stay longer with the company and as a results expertise or skills are not lost.

In terms of future research, the interrelationships between physical and psychological working conditions should be disentangled as physical conditions may be more relevant than psychosocial conditions with respect to RMG workers’ health. Such research would preferably rely on prospective studies.

## Supplementary Information

Below is the link to the electronic supplementary material.

Supplementary file1 (PDF 391 KB)

Supplementary file2 (DOCX 48 KB)

## Data Availability

The data sets generated and analyzed during the current study are not publicly available due to privacy concern, but are available from the corresponding author on reasonable request.
