# Supplementary material for: Work–family conflict, financial issues and their association with self-reported health complaints among ready-made garment workers in Bangladesh: a cross-sectional study
Source: Int Arch Occup Environ Health. 2022 Dec 8;96(4):483–96. doi: 10.1007/s00420-022-01942-9 (PMC9734729; doi:10.1007/s00420-022-01942-9)
Supplement: Supplementary file 1 — Supplementary file1 (PDF 391 KB) [file 420_2022_1942_MOESM1_ESM.pdf]

08 February 2021

Work and health in the ready-made garment sector

English Questionnaire

Independent University of Bangladesh (IUB) Dhaka, Bangladesh

and

Mitra and Associates
Commercial Plot No. 35 (Floor 3rd – 5th), Main Road No. 1
Section 10, Senpara Portoba, Mirpur, Dhaka-1216

Section A: Demographics and Health

I would like to start by asking you some background questions, including some questions about your health.

No.	Question	Response	Skip
1.	Are you a garment worker?<br>*If respondents answer "no", terminate the interview.*	Yes .... 1<br>No.. 2	→ Interv. End
2.	Record sex as observed	Female .... 1<br>Male.................................... 2<br>Other__________________ 3<br>(Specify)	
3.	How old are you?	Years .... □□<br>Don't know 98	→ 5
4.	If you don't know/don't want to tell me your age could you tell me the age range if I read the different options to you?<br>*Read options to the respondents and start out with what seems most appropriate. If respondents are younger than 18 years,* ***terminate the interview.***	Younger than 18 .... 1<br>18-29 .... 2<br>30-39 .... 3<br>40-49 .... 4<br>50-59 .... 5<br>60 or older........................... 6	1 → Interv. End
5.	What was the highest level of schooling you completed?	No formal education .... 1<br>Grade 1-5............................ 2<br>Grade 6-10 .... 3<br>Passed lower secondary exam (matric/SSC) .... 4<br>Passed higher secondary exam (Intermediate/HSC).............. 5<br>Earned bachelor's degree ..... 6<br>Have a post graduate degree . 7	
6.	What is your current marital status?	Married .... 1<br>Separated or divorced .... 2<br>Never married .... 3<br>Husband/wife died 4	3 → 9
7.	Do you have any children?	Yes .... 1<br>No....................................... 2	2 → 9
8.	Are they with you in Dhaka?	Yes .... 1<br>No....................................... 2	
9.	In general, how would you rate your health?	Very good .... 1<br>Good.................................... 2<br>Moderate .... 3<br>Bad .... 4<br>Very Bad............................. 5	

No	Question	Response	Skip
10.	Now I will read out a list of complaints to you and please let me know whether you have been bothered by any of the following complaints in past two months?<br><br>*Read out all the items and tick-off what is answered affirmatively (pause shortly and check with participant after each item)*	Yes No<br>Back pain.................... 1 2<br>Sleeplessness .... 1 2<br>Headache .... 1 2<br>Breathing problems ..... 1 2<br>Cold............................ 1 2<br>Tuberculosis .... 1 2<br>Jaundice...................... 1 2<br>Stomach problem .... 1 2<br>Muscle cramps .... 1 2<br>Eye problems 1 2	
11.	Has a doctor ever told you that you have any of the following conditions?	Yes No<br>Cardiovascular disease 1 2<br>Diabetes...................... 1 2<br>Asthma 1 2	
12.	Do you currently use or smoke any tobacco products, such as betel leaf with jorda, cigarettes, biri, gul, hukkah, dokta, pipes or cigars?	Yes .... 1<br>No 2	
13.	Do you feel that the place where you live is safe regarding the following aspects?	**Yes No**<br>Building safety............... 1 2<br>Safe commuting to work 1 2<br>Infectious diseases prevention ………………1 2<br>Protection against harassment..................... 1 2	

Section B: Occupation

Thank you very much. Next I would like to ask you a few questions about your prior and current work experience in the garment sector.

Now I am going to ask you questions about your job in your current factory.

No.	Question	Response	Skip
14.	When did you first start working in garment factories?	Year, e.g. 2010 .... ☐☐☐☐	
15.	Thinking about (all your jobs) in the last three months, were you mainly working in garment factories?	Yes .... 1<br>No 2	

16.	Thinking about the last three months, how many days have you been unemployed?	Number of days unemployed ....☐☐<br>Not applicable 99	
17.	On average, how many hours do you work per day in your current job?	Hours per day...............☐☐	
18.	For how long have you been working in this factory?	Weeks......................... ☐☐<br>Months......................... ☐☐<br>Years ....☐☐	
19.	Thinking about the last three months, how many days did you <u>NOT</u> work, including weekends and the regular holidays?	Number of days not worked..........................☐☐<br>Not applicable 99	
20.	In the last three months, how many hours overtime did you work?	Total hours of overtime......... ☐☐☐<br>Not applicable 99	
21.	Do you actually get paid for overtime work?	Yes .... 1<br>No 2	
22.	How often do you feel that less working hours are recorded on your time card than you have actually worked?	Never................................. 1<br>Sometimes .... 2<br>Often .... 3<br>Always or almost always 4	
24.	Is your job permanent?	Yes .... 1<br>No .... 2<br>Don't know......................... 3	
25.	Which department do you work at?<br><br>*(Multiple responses are possible)*	Administration/finance .... 1<br>Sampling section .... 2<br>Cutting section .... 3<br>Sewing section .... 4<br>Finishing section .... 5<br>Other (Specify).................... 8	
26.	Which label within that department describes your job best?<br><br>(read out jobs in the suitable section(s); *If respondent does not know the appropriate category, write down his/her own job description*)	<u>Administration/finance</u>:<br>Chairperson...................... 01<br>Managing director .... 02<br>Finance officer .... 03<br>Time keeper .... 04<br>Merchandiser .... 05<br>Cleaner .... 06<br>Body checker .... 07<br>Mechanic .... 08<br>Guard................................ 09<br><u>Sampling section</u>:<br>Sample master................... 11<br>Sample operator 12	

		Sample assistant .... 13<br>Cutting section:<br>Cutting master................... 21<br>Senior cutter .... 22<br>Assistant cutter.................. 23<br>Sewing section:<br>Production Manager .... 31<br>Floor Quality Manager (factory)............................ 32<br>Quality inspector (buying house)............................... 33<br>Line (input) supervisor, production......................... 34<br>Line (output) supervisor, production......................... 35<br>Line supervisor, quality ..... 36<br>Assistant line quality checker .... 37<br>Quality checker at the output table................................. 38<br>Senior machine operator .... 39<br>Junior machine operator..... 40<br>Helpers .... 41<br>Input-man .... 42<br>Finishing section:<br>Finishing head................... 51<br>Ironman .... 52<br>Folder and polyman .... 53<br>Final quality checker .... 54<br>Loader .... 55<br>Other (Specify).................. 98	
27.	Have you already been promoted in this factory to a higher professional rank?	Yes.....................................1<br>No......................................2	
28.	Do you have anyone in the factory from your home district?		
28a.	Supervisor	Yes.....................................1<br>No......................................2<br>Don't know 3	
28b.	Manager	Yes.....................................1<br>No......................................2<br>Don't know 3	

28c.	Factory owner	Yes.....................................1<br>No......................................2<br>Don't know..........................3	

Thank you. Now I am going to read out some statements that people might use to describe their work. We would like to know how you feel regarding these statements. The work related statements that I am going to read out, please compare them with your own job in this factory and tell me one after another whether you agree or disagree with the statements made. Again, I want to emphasize that no one will see how you responded to these questions.

<u>Questions to be asked after each statement is read:</u>

Do you agree or disagree with this statement? Indicate the reply with a circle.

29.	Your job is physically *demanding*.	Yes.....................................1<br>No......................................2<br>Not applicable......................3<br>Does not want to answer 4	
30.	You are under constant time pressure due to a heavy workload.	Yes.....................................1<br>No......................................2<br>Not applicable......................3<br>Does not want to answer 4	
31.	You receive adequate support in difficu lt situations at your workplace.	Yes.....................................1<br>No......................................2<br>Not applicable......................3<br>Does not want to answer 4	
32.	Considering all your efforts your salary is adequate.	Yes.....................................1<br>No......................................2<br>Not applicable......................3<br>Does not want to answer 4	
33.	You receive the recognition you d eserve for your work.	Yes.....................................1<br>No......................................2<br>Not applicable......................3<br>Does not want to answer 4	
34.	Your job promotion prospects are poor.	Yes.....................................1<br>No......................................2<br>Not applicable......................3<br>Does not want to answer 4	

35.	Your job security is poor.	Yes.....................................1<br>No.......................................2<br>Not applicable......................3<br>Does not want to answer 4	
36.	You have very little freedom to decide how you do your work.	Yes.....................................1<br>No.......................................2<br>Not applicable......................3<br>Does not want to answer 4	
37.	<u>*Read only to workers (not to managers)*</u>*:*<br><br>You can trust the information that comes from the management.	Yes.....................................1<br>No.......................................2<br>Not applicable......................3<br>Does not want to answer 4	
38.	<u>*Read only to workers (not to managers)*</u>:<br><br>The management trusts the employees to do their work well.	Yes.....................................1<br>No.......................................2<br>Not applicable......................3<br>Does not want to answer 4	
39.	You are worried about making mistakes at work.	Yes.....................................1<br>No.......................................2<br>Not applicable......................3<br>Does not want to answer 4	
40.	During the last three months, you have been exposed to abusive language at your workplace.	Yes.....................................1<br>No.......................................2<br>Not applicable......................3<br>Does not want to answer 4	
41.	During the last three months, you have been exposed to or witnessed or heard about sexual harassment at your workplace.	Yes.....................................1<br>No.......................................2<br>Not applicable......................3<br>Does not want to answer 4	
42.	You suffer from "manoshik chaap" because of your work	Yes.....................................1<br>No.......................................2<br>Not applicable......................3<br>Does not want to answer 4	
43.	Whenever needed you receive support from your colleagues on the same rank?	Yes.....................................1<br>No.......................................2<br>Not applicable......................3<br>Does not want to answer 4	

44.	Whenever needed you receive support from your supervisor?	Yes.....................................1<br>No.......................................2<br>Not applicable......................3<br>Does not want to answer 4	
45.	You feel bullied by your colleagues on the same rank.	Yes.....................................1<br>No.......................................2<br>Not applicable......................3<br>Does not want to answer 4	
46.	You feel bullied by your supervisor.	Yes.....................................1<br>No.......................................2<br>Not applicable......................3<br>Does not want to answer 4	
47.	Your supervisors do not care about your problems.	Yes.....................................1<br>No.......................................2<br>Not applicable......................3<br>Does not want to answer 4	
48.	Your supervisors take decisions that are free of personal bias.	Yes.....................................1<br>No.......................................2<br>Not applicable......................3<br>Does not want to answer 4	
49.	You think that you could be promoted in this factory one day.	Yes.....................................1<br>No.......................................2<br>Not applicable......................3<br>Does not want to answer ......4<br>Don't know 5	
50.	You think you are currently considered for promotion.	Yes.....................................1<br>No.......................................2<br>Not applicable......................3<br>Does not want to answer ......4<br>Don't know 5	
51.	If one wants to be promoted, one needs to work harder than one's colleagues.	Yes.....................................1<br>No.......................................2<br>Not applicable......................3<br>Does not want to answer 4	
52.	If one wants to be promoted, one needs to be more skilled than one's colleagues.	Yes.....................................1<br>No.......................................2<br>Not applicable......................3<br>Does not want to answer 4	

53.	If one wants to be promoted to a supervisor one must push one's colleagues by all means to meet the production targets.	Yes....................................1<br>No......................................2<br>Not applicable......................3<br>Does not want to answer 4	
54.	You must keep your current job because you need to support your spouse or children financially.	Yes....................................1<br>No......................................2<br>Not applicable......................3<br>Does not want to answer 4	
55.	You must keep your job because you need to financially support other relatives to a significant extent.	Yes....................................1<br>No......................................2<br>Not applicable......................3<br>Does not want to answer 4	
56.	Your family life has disturbed you in doing your job as good as you could do.	Yes....................................1<br>No......................................2<br>Not applicable......................3<br>Does not want to answer 4	
57.	Often you face problems in your family due to your job.	Yes....................................1<br>No......................................2<br>Not applicable......................3<br>Does not want to answer 4	
58.	How long does it take you each working day to travel to and from the factory?<br>(Enter hours or minutes)	Hours .... 1 ☐☐<br>Minutes .... 2 ☐☐	

Next I am going to ask you about your physical working environment.

59.	How crowded is your workplace?	Very low level of crowdedness ....1<br>Low level of crowdedness ....2<br>Average level of crowdedness ....3<br>High level of crowdedness ....4<br>Very high level of crowdedness 5	
60.	What is the level of noise at your workplace?	Very low noise level ....1<br>Low level noise level ....2<br>Average noise level..............3<br>High noise level...................4<br>Very high noise level 5	

61.	Do you feel that your workplace is safe to you regarding the following aspects?	**Yes No**<br>Building safety............... 1 2<br>Machine safety .... 1 2<br>Infectious diseases<br>prevention...................... 1 2<br>Prevention of harassment.1 2	
62.	Were you given safety instructions at your factory (e.g. what to do in case of a fire)?	Yes.....................................1<br>No 2	
63.	Have your ever suffered a physical injury in any factory?	Yes.....................................1<br>No 2	
64.	Do you feel that your safety interests are properly taken into account?	Yes.....................................1<br>No 2	
65.	Is there any workers representative at your workplace?	Yes.....................................1<br>No.....................................2<br>Don't know 3	
66.	Do they represent your interests well?	Yes.....................................1<br>No......................................2<br>Don't know 3	

Section-C: Household Income and Financial Condition

Finally I would like to ask you a few questions about your household and the financial situation.

67.	What was your total personal gross income in the last month?	Total amount<br>of money ..... □□□□□□<br>Not applicable....................99	
68.	In the last month, was your personal gross income less than usual?	Yes.....................................1<br>No......................................2<br>Not applicable....................99	
69.	How many people (including yourself) depend on your wage?	Number of people .... □□<br>Not applicable....................99	
70.	How much of that wage do you hand over to family members?	Total amount<br>of money ..... □□□□□□<br>Not applicable....................99	
71.	How much is monthly room/house rent?	Total amount<br>of money ..... □□□□□□	

	(Including rent, gas, water, electricity etc.)		
72.	Could you call on anyone to support you financially if you lost your job?	Yes.......................................1<br>No...2	
73.	Approximately, if any, how much savings do you have?	Total amount<br>of money ..... □□□□□□	
74.	Approximately, if any, how much is your debt?	Total amount<br>of money ..... □□□□□□	
	Ending time of the interview	Hour .... □□<br>Minute.......................... □□	

Thank you very much for answering these questions. Now we would like to take three thin strands of your hair. Again, we assure that your hair is used for medical research only. It will not be used for any other purpose.

(Again, show a picture of where hair will be taken and show a sample hair strand.)
(Take the strand and thank the respondent for participation.)

75.	Hair Sample Collection	Yes.......................................1<br>No...2	
76.	Number of Hair Strands has been taken	Strand...................................1<br>Strand...................................2<br>Strand...................................3	
	Ending time of hair collection	Hours .... □□<br>Minutes .... □□	
