# Supplementary material for: Work–family conflict, financial issues and their association with self-reported health complaints among ready-made garment workers in Bangladesh: a cross-sectional study
Source: Int Arch Occup Environ Health. 2022 Dec 8;96(4):483–96. doi: 10.1007/s00420-022-01942-9 (PMC9734729; doi:10.1007/s00420-022-01942-9)
Supplement: Supplementary file 2 — Supplementary file2 (DOCX 48 KB) [file 420_2022_1942_MOESM2_ESM.docx]

**Work-family conflict, financial issues and their association with self-reported health complaints among ready-made garment workers in Bangladesh: a cross-sectional study**

International Archives of Occupational and Environmental Health

Annegret Dreher^1^, Rita Yusuf^2^, Hasan Ashraf^3^, Syed A K Shifat Ahmed^2^, Christian Strümpell^4^, Adrian Loerbroks^1^*

1 Institute of Occupational, Social, and Environmental Medicine, Centre for Health and Society, Faculty of Medicine, University of Düsseldorf, Germany
2 International Center for Biotechnology and Health (ICBH), Center for Health Population and Development (CHPD), Independent University, Bangladesh
3 Department of Anthropology, Jahangirnagar University, Bangladesh
4 Institute of Social and Cultural Anthropology, University of Hamburg, Germany

*corresponding author; mail: adrian.loerbroks@uni-duesseldorf.de

**Supplementary Table: The association of work-family conflict and financial matters with self-reported health complaints among n= 797 female ready-made garment workers**

|  | Poor SRH | | Back pain | | Sleeplessness | | Headache | | Cold | |
| --- | --- | --- | --- | --- | --- | --- | --- | --- | --- | --- |
|  | PR | 95% CI | PR | 95% CI | PR | 95% CI | PR | 95% CI | PR | 95% CI |
| Family life has disturbed job (yes vs. no) | **1.27** | **1.04-1.55** | 1.13 | 0.96-1.33 | **1.39** | **1.09-1.78** | 1.00 | 0.88-1.13 | 1.12 | 0.96-1.31 |
| Problems in family due to job (yes vs. no) | 1.05 | 0.84-1.32 | 1.18 | 1.00-1.39 | 1.21 | 0.93-1.58 | 0.99 | 0.87-1.13 | 1.10 | 0.94-1.30 |
| Financial support available (yes vs. no) | **0.76** | **0.63-0.92** | 0.91 | 0.79-1.05 | **0.77** | **0.60-0.98** | 0.93 | 0.85-1.02 | 0.92 | 0.81-1.05 |
| Any savings (yes vs. no) | 0.91 | 0.75-1.11 | 1.15 | 1.00-1.31 | 0.94 | 0.74-1.20 | **1.14** | **1.04-1.25** | 1.06 | 0.93-1.21 |
| Any debt (yes vs. no) | 1.18 | 1.00-1.40 | **1.15** | **1.01-1.31** | 1.19 | 0.96-1.47 | 1.10 | 1.01-1.20 | 1.12 | 0.99-1.26 |
| Amount of savings  Less than 1 monthly salary  1-2 monthly salaries  3 or more monthly salaries | Ref. 0.90 0.95 | Ref. 0.65-1.25 0.73-1.23 | Ref. 1.13 1.19 | Ref. 0.92-1.39 1.00-1.42 | Ref. 1.27 0.95 | Ref. 0.92-1.76 0.68-1.33 | Ref. 1.09 **1.14** | Ref. 0.94-1.27 **1.02-1.28** | Ref. 1.09 0.96 | Ref. 0.89-1.33 0.79-1.17 |
| Amount of debt  Less than 1 monthly salary  1-2 monthly salaries  3 or more monthly salaries | Ref. 0.95 1.10 | Ref. 0.72-1.25 0.90-1.34 | Ref. 0.98 1.14 | Ref. 0.79-1.20 0.98-1.33 | Ref. 1.09 1.07 | Ref. 0.79-1.51 0.83-1.39 | Ref. 1.06 **1.15** | Ref. 0.92-1.21 **1.03-1.27** | Ref. 0.94 **1.23** | Ref. 0.77-1.16 **1.07-1.42** |
| Number of persons depending on wage | 0.99 | 0.93-1.05 | 1.00 | 0.95-1.05 | 1.02 | 0.95-1.10 | 1.00 | 0.97-1.03 | 1.00 | 0.96-1.05 |
| Financial support of children or spouse (yes vs. no) | 1.22 | 0.77-1.92 | 1.24* | 0.84-1.83* | 1.15 | 0.67-2.00 | 1.03 | 0.81-1.29 | 0.97 | 0.72-1.29 |
| Financial support of other relatives (yes vs. no) | 0.84 | 0.71-1.01 | 1.02 | 0.88-1.17 | 0.97 | 0.78-1.21 | 0.93 | 0.84-1.03 | 0.92 | 0.80-1.05 |

Poisson regression results in form of prevalence ratios (PR) with respective 95% confidence intervals (CI). Adjusted for age, marital status, education and tobacco use. Significant findings highlighted in bold.

* Significant in interaction analyses

**Supplementary Table (continued): The association of work-family conflict and financial matters with self-reported health complaints among n= 797 female ready-made garment workers**

|  | Jaundice | | Stomach problems | | Muscle cramp | | Eye problems | |
| --- | --- | --- | --- | --- | --- | --- | --- | --- |
|  | PR | 95% CI | PR | 95% CI | PR | 95% CI | PR | 95% CI |
| Family life has disturbed job (yes vs. no) | **0.96*** | **0.77-1.19*** | 1.06 | 0.79-1.41 | **1.40** | **1.18-1.66** | 1.07 | 0.76-1.50 |
| Problems in family due to job (yes vs. no) | 0.97 | 0.77-1.21 | 1.04 | 0.77-1.40 | **1.42** | **1.19-1.69** | 1.09 | 0.77-1.54 |
| Financial support available (yes vs. no) | 0.91 | 0.77-1.08 | 0.85 | 0.68-1.08 | 0.89 | 0.76-1.06 | 0.77 | 0.58-1.02 |
| Any savings (yes vs. no) | 0.88 | 0.73-1.06 | 0.90 | 0.70-1.16 | 1.12 | 0.95-1.32 | 0.89 | 0.66-1.19 |
| Any debt (yes vs. no) | 1.11 | 0.95-1.30 | **1.45** | **1.18-1.80** | **1.18** | **1.01-1.38** | 1.22 | 0.96-1.56 |
| Amount of savings  Less than 1 monthly salary  1-2 monthly salaries  3 or more monthly salaries | Ref. 0.75 1.00 | Ref. 0.53-1.07 0.79-1.27 | Ref. 0.73 0.88 | Ref. 0.46-1.16 0.62-1.25 | Ref. 1.18 0.97 | Ref. 0.93-1.49 0.76-1.25 | Ref. 0.73 1.07 | Ref. 0.45-1.20 0.74-1.55 |
| Amount of debt  Less than 1 monthly salary  1-2 monthly salaries  3 or more monthly salaries | Ref. 1.04 1.18 | Ref. 0.81-1.34 0.98-1.43 | Ref. 1.29 **1.58** | Ref. 0.93-1.78 **1.24-2.02** | Ref. 1.00 **1.24** | Ref. 0.77-1.29 **1.04-1.48** | Ref. 1.03 **1.38** | Ref. 0.68-1.57 **1.04-1.83** |
| Number of persons depending on wage | 0.97 | 0.91-1.03 | 1.06 | 0.98-1.14 | 0.98 | 0.93-1.04 | **1.13** | **1.04-1.23** |
| Financial support of children or spouse (yes vs. no) | 0.99 | 0.67-1.46 | 0.93 | 0.57-1.51 | **1.58*** | **0.95-2.62*** | 1.88 | 0.93-3.79 |
| Financial support of other relatives (yes vs. no) | 1.02 | 0.87-1.21 | 0.92 | 0.73-1.16 | 1.07 | 0.91-1.27 | 1.28 | 0.98-1.66 |

Poisson regression results in form of prevalence ratios (PR) with respective 95% confidence intervals (CI). Adjusted for age, sex, marital status, education and tobacco use. Significant findings highlighted in bold.

* Significant in interaction analyses

**Supplementary Table: The association of work-family conflict and financial matters with self-reported health complaints among n= 321 male ready-made garment workers**

|  | Poor SRH | | Back pain | | Sleeplessness | | Headache | | Cold | |
| --- | --- | --- | --- | --- | --- | --- | --- | --- | --- | --- |
|  | PR | 95% CI | PR | 95% CI | PR | 95% CI | PR | 95% CI | PR | 95% CI |
| Family life has disturbed job (yes vs. no) | **1.69** | **1.15-2.48** | 1.24 | 0.89-1.73 | 1.12 | 0.70-1.78 | 1.10 | 0.87-1.41 | 1.20 | 0.91-1.59 |
| Problems in family due to job (yes vs. no) | 1.19 | 0.73-1.93 | 1.08 | 0.71-1.64 | 1.06 | 0.62-1.81 | 1.19 | 0.93-1.52 | 1.17 | 0.85-1.62 |
| Financial support available (yes vs. no) | 0.73 | 0.49-1.10 | **0.70** | **0.51-0.96** | **0.62** | **0.41-0.95** | **0.77** | **0.62-0.96** | **0.76** | **0.58-0.98** |
| Any savings (yes vs. no) | 1.00 | 0.67-1.50 | 0.96 | 0.70-1.32 | 1.10 | 0.75-1.60 | 0.95 | 0.76-1.18 | 0.88 | 0.67-1.17 |
| Any debt (yes vs. no) | 0.88 | 0.62-1.26 | 1.21 | 0.93-1.57 | 1.28 | 0.92-1.79 | 1.07 | 0.89-1.28 | 1.23 | 0.99-1.53 |
| Amount of savings  Less than 1 monthly salary  1-2 monthly salaries  3 or more monthly salaries | Ref. 1.50 0.28 | Ref. 0.94-2.40 0.08-1.04 | Ref. 0.67 0.87 | Ref. 0.37-1.20 0.51-1.50 | Ref. 1.03 0.56 | Ref. 0.60-1.78 0.23-1.37 | Ref. 0.77 0.88 | Ref. 0.51-1.15 0.60-1.30 | Ref. 1.01 0.84 | Ref. 0.69-1.48 0.53-1.34 |
| Amount of debt  Less than 1 monthly salary  1-2 monthly salaries  3 or more monthly salaries | Ref. 1.11 0.90 | Ref. 0.70-1.75 0.54-1.49 | Ref. **1.45** 1.20 | Ref. **1.07-1.96** 0.84-1.73 | Ref. 1.13 **1.71** | Ref. 0.71-1.81 **1.15-2.55** | Ref. 1.09 0.97 | Ref. 0.86-1.38 0.75-1.26 | Ref. 1.13 1.16 | ref. 0.85-1.51 0.86-1.56 |
| Number of persons depending on wage | 1.00 | 0.89-1.13 | 1.02 | 0.94-1.12 | 1.00 | 0.89-1.12 | 0.99 | 0.93-1.05 | 0.96 | 0.89-1.04 |
| Financial support of children or spouse (yes vs. no) | 2.67 | 0.56-12.70 | 0.46* | 0.13-1.59* | 1.45 | 0.38-5.52 | **0.45** | **0.27-0.74** | 0.42 | 0.16-1.34 |
| Financial support of other relatives (yes vs. no) | 1.11 | 0.73-1.70 | 1.03 | 0.75-1.43 | 1.08 | 0.71-1.63 | 0.86 | 0.70-1.06 | 0.92 | 0.71-1.19 |

Poisson regression results in form of prevalence ratios (PR) with respective 95% confidence intervals (CI). Adjusted for age, marital status, education and tobacco use. Significant findings highlighted in bold.

* Significant in interaction analyses

**Supplementary Table (continued): The association of work-family conflict and financial matters with self-reported health complaints among n= 321 male ready-made garment workers**

|  | Jaundice | | Stomach problems | | Muscle cramp | | Eye problems | |
| --- | --- | --- | --- | --- | --- | --- | --- | --- |
|  | PR | 95% CI | PR | 95% CI | PR | 95% CI | PR | 95% CI |
| Family life has disturbed job (yes vs. no) | **1.50*** | **1.11-2.05*** | 0.97 | 0.55-1.72 | **1.44** | **1.02-2.03** | 1.55 | 0.89-2.70 |
| Problems in family due to job (yes vs. no) | 1.41 | 1.00-2.00 | **1.69** | **1.05-2.73** | 1.28 | 0.84-1.95 | 1.56 | 0.86-2.85 |
| Financial support available (yes vs. no) | 0.83 | 0.62-1.11 | **0.61** | **0.38-0.98** | 0.77 | 0.54-1.09 | **0.51** | **0.28-0.93** |
| Any savings (yes vs. no) | 0.72 | 0.50-1.04 | 1.19 | 0.77-1.84 | 0.96 | 0.67-1.38 | 0.75 | 0.41-1.39 |
| Any debt (yes vs. no) | 1.23 | 0.95-1.59 | 1.16 | 0.79-1.72 | 1.28 | 0.95-1.72 | 1.46 | 0.93-2.30 |
| Amount of savings  Less than 1 monthly salary  1-2 monthly salaries  3 or more monthly salaries | Ref. 1.02 0.51 | Ref. 0.66-1.59 0.23-1.10 | Ref. 1.21 1.07 | Ref. 0.64-2.28 0.52-2.21 | Ref. 1.27 0.86 | Ref. 0.82-1.96 0.45-1.65 | Ref. 0.69 0.66 | Ref. 0.26-1.81 0.25-1.78 |
| Amount of debt  Less than 1 monthly salary  1-2 monthly salaries  3 or more monthly salaries | Ref. **1.51** 1.25 | Ref. **1.11-2.05** 0.88-1.76 | Ref. 1.24 0.90 | Ref. 0.76-2.02 0.50-1.63 | Ref. 1.25 1.43 | Ref. 0.84-1.84 0.98-2.09 | Ref. 1.64 1.58 | Ref. 0.93-2.91 0.89-2.80 |
| Number of persons depending on wage | 1.01 | 0.93-1.10 | 0.98 | 0.86-1.12 | 0.96 | 0.86-1.07 | 1.07 | 0.93-1.24 |
| Financial support of children or spouse (yes vs. no) | 3.98 | 0.68-23.23 | **0.16** | **0.07-0.34** | **0.23*** | **0.12-0.46*** | 0.31 | 0.06-1.60 |
| Financial support of other relatives (yes vs. no) | 0.96 | 0.70-1.30 | 0.67 | 0.43-1.04 | 1.28 | 0.87-1.87 | 0.79 | 0.47-1.31 |

Poisson regression results in form of prevalence ratios (PR) with respective 95% confidence intervals (CI). Adjusted for age, marital status, education and tobacco use. Significant findings highlighted in bold.

* Significant in interaction analyses

**Supplementary Table: The association of work-family conflict and financial matters with self-reported health complaints among n=610 ready-made garment workers aged 18-25 (younger group according to median split)**

|  | Poor SRH | | Back pain | | Sleeplessness | | Headache | | Cold | |
| --- | --- | --- | --- | --- | --- | --- | --- | --- | --- | --- |
|  | PR | 95% CI | PR | 95% CI | PR | 95% CI | PR | 95% CI | PR | 95% CI |
| Family life has disturbed job (yes vs. no) | 1.24 | 0.92-1.66 | 1.08 | 0.86-1.35 | **1.43** | **1.04-1.98** | 1.03 | 0.88-1.19 | 1.06 | 0.87-1.30 |
| Problems in family due to job (yes vs. no) | 0.91 | 0.63-1.31 | 1.21 | 0.97-1.50 | 1.09 | 0.74-1.61 | 1.02 | 0.87-1.20 | 1.04 | 0.84-1.29 |
| Financial support available (yes vs. no) | **0.71** | **0.56-0.91** | 0.84 | 0.71-1.00 | **0.72** | **0.54-0.96** | **0.84*** | **0.75-0.94*** | **0.85** | **0.73-0.99** |
| Any savings (yes vs. no) | 0.87 | 0.66-1.16 | 1.16 | 0.97-1.39 | 0.90 | 0.65-1.24 | **1.17** | **1.05-1.31** | 0.95 | 0.79-1.13 |
| Any debt (yes vs. no) | **1.33** | **1.05-1.68** | **1.25** | **1.06-1.48** | **1.43** | **1.09-1.89** | 1.09 | 0.97-1.22 | 1.06 | 0.91-1.24 |
| Amount of savings  Less than 1 monthly salary  1-2 monthly salaries  3 or more monthly salaries | Ref. 1.15 0.64 | Ref. 0.77-1.72 0.40-1.04 | Ref. 1.20 1.03 | Ref. 0.93-1.55 0.79-1.36 | Ref. 1.22 0.75 | Ref. 0.80-1.86 0.44-1.31 | Ref. 1.06 1.16 | Ref. 0.86-1.29 1.00-1.34 | Ref. 1.00 0.83 | Ref. 0.76-1.32 0.62-1.11 |
| Amount of debt  Less than 1 monthly salary  1-2 monthly salaries  3 or more monthly salaries | Ref. 1.01 1.29 | Ref. 0.69-1.48 0.94-1.78 | Ref.* 1.03* **1.40*** | Ref.* 0.79-1.36* **1.14-1.71*** | Ref. 1.15 **1.49** | Ref. 0.78-1.72 **1.03-2.17** | Ref. 0.95 **1.22** | Ref. 0.78-1.16 **1.08-1.38** | Ref. 0.89 1.22 | Ref. 0.68-1.15 1.00-1.48 |
| Number of persons depending on wage | 0.98 | 0.90-1.07 | 1.01 | 0.95-1.07 | 0.98 | 0.89-1.08 | 0.99 | 0.95-1.03 | 0.99 | 0.94-1.05 |
| Financial support of children or spouse (yes vs. no) | 2.23 | 0.88-5.68 | 1.15 | 0.83-2.53 | 1.86 | 0.72-4.81 | 1.09 | 0.79-1.49 | 0.92 | 0.64-1.32 |
| Financial support of other relatives (yes vs. no) | 0.89 | 0.67-1.19 | 1.10 | 0.89-1.35 | 0.99 | 0.71-1.38 | 0.93 | 0.82-1.07 | 0.97 | 0.80-1.16 |

Poisson regression results in form of prevalence ratios (PR) with respective 95% confidence intervals (CI). Adjusted for age, marital status, education and tobacco use. Significant findings highlighted in bold.

* Significant in interaction analyses

**Supplementary Table (continued): The association of work-family conflict and financial matters with self-reported health complaints among n=610 ready-made garment workers aged 18-25 (younger group according to median split)**

|  | Jaundice | | Stomach problems | | Muscle cramp | | Eye problems | |
| --- | --- | --- | --- | --- | --- | --- | --- | --- |
|  | PR | 95% CI | PR | 95% CI | PR | 95% CI | PR | 95% CI |
| Family life has disturbed job (yes vs. no) | 1.08 | 0.84-1.40 | 0.95 | 0.64-1.39 | **1.48** | **1.18-1.86** | 1.10 | 0.68-1.77 |
| Problems in family due to job (yes vs. no) | 0.83* | 0.60-1.14* | 1.12 | 0.76-1.64 | **1.54** | **1.22-1.95** | 1.38 | 0.87-2.19 |
| Financial support available (yes vs. no) | 0.86 | 0.71-1.04 | **0.70*** | **0.53-0.92*** | 0.85 | 0.70-1.05 | **0.67** | **0.47-0.96** |
| Any savings (yes vs. no) | 0.80 | 0.63-1.02 | 1.06 | 0.78-1.44 | 1.11 | 0.89-1.39 | 0.94 | 0.62-1.41 |
| Any debt (yes vs. no) | 1.18 | 0.97-1.42 | **1.42** | **1.09-1.86** | 1.23 | 1.00-1.50 | **1.47** | **1.04-2.07** |
| Amount of savings  Less than 1 monthly salary  1-2 monthly salaries  3 or more monthly salaries | Ref. 0.70 0.90 | Ref. 0.45-1.10 0.65-1.24 | Ref. 1.17 0.98 | Ref. 0.73-1.86 0.62-1.54 | Ref. 1.27 1.03 | Ref. 0.91-1.77 0.74-1.44 | Ref. 0.63 1.03 | Ref. 0.27-1.43 0.59-1.79 |
| Amount of debt  Less than 1 monthly salary  1-2 monthly salaries  3 or more monthly salaries | Ref. **1.39** 1.21 | Ref. **1.08-1.79** 0.92-1.58 | Ref. 1.18 **1.60** | Ref. 0.78-1.80 **1.14-2.25** | Ref. 1.07 1.25 | Ref. 0.77-1.48 0.94-1.66 | Ref. 1.30 **1.68** | Ref. 0.76-2.20 **1.06-2.65** |
| Number of persons depending on wage | 0.99 | 0.92-1.06 | 0.98* | 0.89-1.07* | 0.98* | 0.91-1.05* | **1.16** | **1.04-1.30** |
| Financial support of children or spouse (yes vs. no) | 0.85* | 0.56-1.31* | 0.91* | 0.46-1.83* | 1.20* | 0.71-2.04* | **3.30** | **1.01-10.81** |
| Financial support of other relatives (yes vs. no) | 1.04 | 0.82-1.32 | 0.85 | 0.61-1.20 | 1.11 | 0.86-1.43 | **1.76** | **1.08-2.89** |

Poisson regression results in form of prevalence ratios (PR) with respective 95% confidence intervals (CI). Adjusted for age, marital status, education and tobacco use. Significant findings highlighted in bold.

* Significant in interaction analyses

**Supplementary Table: The association of work-family conflict and financial matters with self-reported health complaints among n=508 ready-made garment workers aged 26 and above (older group according to median split)**

|  | Poor SRH | | Back pain | | Sleeplessness | | Headache | | Cold | |
| --- | --- | --- | --- | --- | --- | --- | --- | --- | --- | --- |
|  | PR | 95% CI | PR | 95% CI | PR | 95% CI | PR | 95% CI | PR | 95% CI |
| Family life has disturbed job (yes vs. no) | **1.44** | **1.16-1.78** | 1.22 | 1.00-1.49 | 1.14 | 0.85-1.52 | 1.02 | 0.87-1.20 | 1.19 | 0.99-1.43 |
| Problems in family due to job (yes vs. no) | 1.22 | 0.96-1.55 | 1.11 | 0.89-1.39 | 1.15 | 0.85-1.56 | 1.06 | 0.90-1.25 | 1.16 | 0.96-1.40 |
| Financial support available (yes vs. no) | 0.82 | 0.64-1.05 | 0.89 | 0.73-1.08 | 0.78 | 0.58-1.06 | 1.01* | 0.88-1.16* | 0.93 | 0.78-1.12 |
| Any savings (yes vs. no) | 0.98 | 0.79-1.22 | 1.03 | 0.86-1.24 | 1.04 | 0.80-1.34 | 1.01 | 0.89-1.16 | 1.08 | 0.92-1.27 |
| Any debt (yes vs. no) | 0.97 | 0.80-1.17 | 1.09 | 0.93-1.28 | 1.03 | 0.82-1.29 | 1.10 | 0.98-1.24 | **1.22** | **1.05-1.42** |
| Amount of savings  Less than 1 monthly salary  1-2 monthly salaries  3 or more monthly salaries | Ref. 0.91 1.00 | Ref. 0.63-1.31 0.75-1.35 | Ref. 0.86 1.20 | Ref. 0.62-1.17 0.97-1.49 | Ref. 1.11 1.01 | Ref. 0.77-1.60 0.70-1.47 | Ref. 0.96 1.00 | Ref. 0.77-1.19 0.82-1.21 | Ref. 1.09 1.08 | Ref. 0.86-1.38 0.86-1.36 |
| Amount of debt  Less than 1 monthly salary  1-2 monthly salaries  3 or more monthly salaries | Ref. 0.96 0.96 | Ref. 0.71-1.30 0.77-1.20 | Ref.* 1.16* 1.04* | Ref.* 0.93-1.44* 0.86-1.25* | Ref. 0.98 1.07 | Ref. 0.69-1.39 0.82-1.39 | Ref. **1.18** 1.05 | Ref. **1.02-1.36** 0.91-1.21 | Ref. 1.09 1.23 | Ref. 0.88-1.36 1.04-1.46 |
| Number of persons depending on wage | 1.00 | 0.93-1.08 | 1.02 | 0.96-1.08 | 1.04 | 0.97-1.13 | 1.00 | 0.96-1.05 | 0.99 | 0.94-1.05 |
| Financial support of children or spouse (yes vs. no) | 0.73 | 0.46-1.15 | 0.86 | 0.56-1.31 | 0.75 | 0.42-1.33 | 0.81 | 0.63-1.03 | 0.84 | 0.58-1.23 |
| Financial support of other relatives (yes vs. no) | 0.86 | 0.71-1.04 | 0.97 | 0.82-1.15 | 0.98 | 0.78-1.24 | 0.92 | 0.81-1.03 | 0.88 | 0.76-1.03 |

Poisson regression results in form of prevalence ratios (PR) with respective 95% confidence intervals (CI). Adjusted for age, marital status, education and tobacco use. Significant findings highlighted in bold.

* Significant in interaction analyses

**Supplementary Table (continued): The association of work-family conflict and financial matters with self-reported health complaints among n=508 ready-made garment workers aged 26 and above (older group according to median split)**

|  | Jaundice | | Stomach problems | | Muscle cramp | | Eye problems | |
| --- | --- | --- | --- | --- | --- | --- | --- | --- |
|  | PR | 95% CI | PR | 95% CI | PR | 95% CI | PR | 95% CI |
| Family life has disturbed job (yes vs. no) | 1.09 | 0.85-1.39 | 1.09 | 0.78-1.52 | **1.39** | **1.13-1.70** | 1.21 | 0.84-1.73 |
| Problems in family due to job (yes vs. no) | **1.33*** | **1.06-1.68*** | 1.14 | 0.80-1.61 | **1.30** | **1.04-1.63** | 1.02 | 0.68-1.53 |
| Financial support available (yes vs. no) | 0.95 | 0.76-1.20 | 1.00* | 0.74-1.35* | 0.91 | 0.73-1.15 | 0.75 | 0.52-1.07 |
| Any savings (yes vs. no) | 0.86 | 0.68-1.07 | 0.86 | 0.63-1.17 | 1.06 | 0.86-1.29 | 0.81 | 0.58-1.14 |
| Any debt (yes vs. no) | 1.10 | 0.92-1.33 | **1.30** | **1.01-1.69** | 1.18 | 0.98-1.42 | 1.16 | 0.89-1.52 |
| Amount of savings  Less than 1 monthly salary  1-2 monthly salaries  3 or more monthly salaries | Ref. 0.94 0.89 | Ref. 0.66-1.33 0.65-1.23 | Ref. 0.58 0.89 | Ref. 0.31-1.07 0.57-1.38 | Ref. 1.19 0.89 | Ref. 0.90-1.57 0.64-1.25 | Ref. 0.78 1.01 | Ref. 0.46-1.31 0.66-1.53 |
| Amount of debt  Less than 1 monthly salary  1-2 monthly salaries  3 or more monthly salaries | Ref. 1.02 1.13 | Ref. 0.76-1.37 0.92-1.39 | Ref. 1.35 1.27 | Ref. 0.95-1.93 0.95-1.70 | Ref. 1.04 **1.28** | Ref. 0.78-1.38 **1.05-1.57** | Ref. 1.16 1.30 | Ref. 0.76-1.76 0.97-1.75 |
| Number of persons depending on wage | 0.96 | 0.90-1.03 | 1.08* | 0.99-1.18* | 0.98* | 0.91-1.05* | **1.10** | **1.01-1.21** |
| Financial support of children or spouse (yes vs. no) | 1.74* | 0.75-4.07* | 0.75* | 0.38-1.46* | 1.72* | 0.73-4.07* | 0.98 | 0.45-2.16 |
| Financial support of other relatives (yes vs. no) | 1.03 | 0.85-1.24 | 0.89 | 0.68-1.16 | 1.09 | 0.90-1.32 | 0.98 | 0.74-1.31 |

Poisson regression results in form of prevalence ratios (PR) with respective 95% confidence intervals (CI). Adjusted for age, marital status, education and tobacco use. Significant findings highlighted in bold.

* Significant in interaction analyses
